# Fatty acid composition of lipid fractions in white- and brown-like adipocytes derived from human mesenchymal stem cells

**DOI:** 10.1080/21623945.2025.2566481

**Published:** 2025-10-01

**Authors:** Khadijeh Abbasi, Amir Mehdizadeh, Hamed Hamishehkar, Mohammad Nouri, Masoud Darabi

**Affiliations:** a Drug Applied Research Center, Tabriz University of Medical Sciences, Tabriz, Iran; b Department of Biochemistry and Clinical Laboratories, Faculty of Medicine, Tabriz University of Medical Sciences, Tabriz, Iran; c Hematology and Oncology Research Center, Tabriz University of Medical Sciences, Tabriz, Iran; d Research Center of New Material and Green Chemistry, Khazar University, Baku, Azerbaijan; e Division of Experimental Oncology, Department of Hematology and Oncology, University Medical Center Schleswig-Holstein (UKSH), Campus Lübeck, Lübeck, Germany; f University Medical Center Schleswig-Holstein (UKSH), University Cancer Center Schleswig-Holstein (UCCSH), Campus Lübeck, Lübeck, Germany

**Keywords:** Lipid metabolism, mitochondria, phospholipids, thermogenesis, triglycerides

## Abstract

White and brown adipocytes differ markedly in lipid composition and metabolic function. White adipocytes primarily serve as energy storage depots, whereas brown adipocytes are mitochondria-rich and specialized for thermogenesis. However, the lipidomic profiles of white-like (WLAs) and brown-like adipocytes (BLAs) differentiated from human mesenchymal stem cells (MSCs) remain incompletely characterized. Human adipose-derived MSCs were differentiated into WLAs and BLAs. Lipid fractions were isolated and analysed by gas-liquid chromatography. Fatty acid composition data were used to calculate indices of stearoyl-CoA desaturase-1 (SCD1) activity, elongation, and ω6 synthesis. Compared to MSCs, BLAs showed consistently elevated oleate (≥4.2-fold) and stearate (≥2.3-fold), along with reduced palmitate (≤−20%) and linoleate (≤−28%) across phospholipid, triglyceride, and free fatty acid fractions. WLAs versus MSCs showed similar trends, with oleate increasing up to 15-fold and palmitate decreasing by 67–82% depending on the lipid class. SCD1 activity and elongation indices were elevated in WLAs (SCD1: up to 4.7-fold; elongation: up to 28-fold). The ω6 synthesis index was also increased in triglyceride and free fatty acid fractions of WLAs (≥3.3-fold), but markedly suppressed in BLAs (≤−88.7%). WLAs and BLAs differentiated from MSCs exhibit distinct lipid profiles and inferred enzymatic activity patterns, reflecting their respective capacities for lipid storage and metabolic flexibility. These findings provide a foundation for future translational research aimed at targeting adipose tissue in obesity and metabolic diseases.

## GRAPHICAL ABSTRACT

**Figure:**
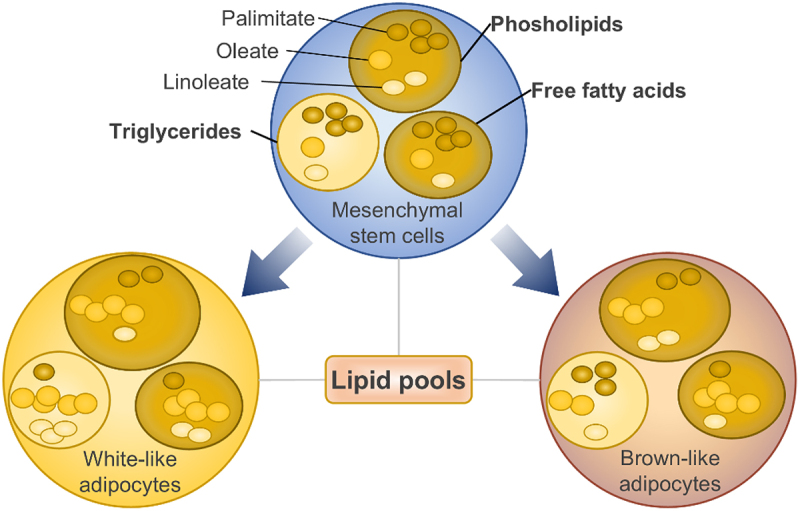

## 1. Introduction

Adipose tissue plays a central role in energy homoeostasis and metabolic regulation and is primarily composed of two functionally distinct cell types: white and brown adipocytes [1]. White adipocytes are characterized by a single large lipid droplet and primarily function as long-term energy storage depots. In contrast, brown adipocytes contain a high density of mitochondria and multilocular lipid droplets, which together support their thermogenic capacity. This is driven by mitochondrial β-oxidation of fatty acids, with CPT1b mediating their import into mitochondria, and the resulting acetyl-CoA entering the tricarboxylic acid cycle via citrate synthase. The energy from this breakdown is dissipated as heat by uncoupling protein 1 (UCP1), which bypasses the typical ATP-synthesis pathway [2,3]. An imbalance between white and brown adipose tissue function, manifested as adipocyte ‘whitening’ or ‘browning’, has been implicated in the pathogenesis of obesity, type 2 diabetes, cancer and related metabolic disorders [1,4].

Beyond morphological and functional distinctions, white and brown adipocytes exhibit differing lipid metabolic profiles, including distinct fatty acid compositions that reflect their specialized physiological roles [5–8]. Notably, alterations in the fatty acid profile of adipose tissue, such as increased chain length or higher levels of monounsaturated fatty acids, have been associated with obesity and insulin resistance in both human and animal studies [9,10].

Human mesenchymal stem cells (MSCs) can be differentiated into white- (WLAs) or brown-like adipocytes (BLAs), providing robust in vitro models to investigate adipocyte biology [11]. Beyond their experimental utility, MSCs are also among the most promising candidate cell sources for generating autologous adipocytes for future cell-based therapies targeting metabolic disorders and adipose tissue dysfunction. However, a comprehensive understanding of the specific lipid content of MSC differentiated adipocytes, which is crucial for the cellular processes governing energy metabolism and adipose tissue phenotype, remains unclear.

In this study, we aimed to characterize and compare the fatty acid composition of phospholipid, triglyceride, and free fatty acid fractions in MSC-derived WLAs and BLAs. By analysing these lipid fractions, we have gained better understanding of the metabolic distinctions between these adipocyte phenotypes and their potential relevance to adipose tissue dysfunction in metabolic disease.

## 2. Material and methods

### 2.1. Isolation of MSCs from human adipose tissue

MSCs were isolated according to our previously established protocol [12]. Subcutaneous adipose tissue was obtained from a healthy donor undergoing elective abdominal lipectomy, with informed consent. The study was conducted in accordance with the Declaration of Helsinki and approved by the Institutional Ethics Committee of Tabriz University of Medical Sciences (IR.TBZMED.REC.1401.198).

The tissue was washed with phosphate-buffered saline, transferred onto a sterile glass plate, and finely minced using a surgical blade. The minced tissue was digested with 2% type I collagenase and incubated at 37°C. Enzymatic digestion was neutralized by adding an equal volume of glucose-supplemented Dulbecco’s Modified Eagle Medium (DMEM; Gibco, USA) containing 10% foetal bovine serum (FBS; AnaCell, Iran).

The digested tissue was centrifuged at 350 g for 10 minutes. The supernatant was discarded, and the cell pellet was resuspended and washed twice with DMEM. Red blood cells were lysed using ammonium chloride (NH₄Cl) solution. The remaining cells were cultured in DMEM supplemented with 10% FBS and 1% penicillin-streptomycin. Cells were seeded into T25 flasks and incubated at 37°C in a humidified atmosphere containing 5% CO₂. The medium was changed every 48 hours until 80% confluence was reached. MSCs were characterized by flow cytometry for their surface marker profile (CD44^+^, CD90^+^, CD73^+^, CD105^+^, CD31^−^, CD34^−^, CD45^−^) and their adipogenic differentiation capacity, as previously reported by our group [13].

### 2.2. In vitro differentiation of MSCs into white- and brown-like adipocytes

Differentiation protocols were adapted from established methods [14].

#### 2.2.1. Differentiation into brown-like adipocytes

MSCs were seeded in 6-well plates and cultured at 37°C for 24 hours. Upon reaching ~ 80% confluence (day 0 of differentiation), the medium was replaced with a browning induction cocktail containing low-glucose DMEM (DMEM-LG), 10% FBS, 1 µg/mL insulin, 0.2 µM dexamethasone, 0.2 nM triiodothyronine (T3), 1 µM rosiglitazone, and 0.5 mM 3-isobutyl-1-methylxanthine (IBMX). Differentiation was carried out over 14 days, with media changes every 3 days.

#### 2.2.2. Differentiation into white-like adipocytes

To induce white-like differentiation, MSCs were cultured in DMEM-LG supplemented with 10% FBS, 0.2 nM T3, 1 μM dexamethasone, 0.5 mM IBMX, and 1 µg/mL insulin. The medium was replaced every 3 days for a total of 14 days. Undifferentiated MSCs maintained in standard growth medium with 10% FBS served as the control group.

### 2.3. Characterization of differentiation

#### 2.3.1. Quantification of UCP1 and CPT1b mRNA expression

Total RNA was extracted from MSCs, WLAs, and BLAs using YTzol reagent (Yekta Tajhiz Azma, Iran) following the manufacturer’s instructions. RNA purity and concentration were assessed spectrophotometrically, and 200 ng of RNA was used for cDNA synthesis using a commercial kit (Yekta Tajhiz Azma, Iran). The resulting cDNA was stored at − 20°C.

Quantitative PCR was performed using a MIC real-time PCR system (BioMolecular Systems, Australia) with SYBR Green PCR Master Mix (Yekta Tajhiz Azma, Iran). The relative expression of UCP1 was calculated using the 2^–ΔΔCT method, with GAPDH as the housekeeping gene. Primer sequences were designed using NCBI Primer-BLAST and validated using Oligo7 software (Supplementary Table S1).

#### 2.3.2. Detection of UCP1 and CPT1b protein amount

Cells were lysed in RIPA buffer (DNAbiotech, Iran), and lysates were centrifuged at 12,000 g for 10 minutes at 4°C. Protein concentrations were measured using the Bradford assay. Equal amounts (20 µg) of total protein were mixed with sample buffer, separated by sodium dodecyl sulphate-polyacrylamide gel electrophoresis, and transferred to polyvinylidene difluoride membranes. Membranes were blocked with 5% skim milk and incubated overnight at 4°C with primary antibodies against UCP1 and GAPDH (Santa Cruz Biotechonolgy, USA). After washing, membranes were incubated with horseradish peroxidase-conjugated secondary antibodies for 1 hour at room temperature. Bands were visualized using luminol-based chemiluminescence reagents (Santa Cruz Biotechonolgy) and quantified using ImageJ software (NIH, USA).

#### 2.3.3. Citrate synthase activity measurement

Citrate synthase activity was measured to assess mitochondrial activity. The assay was based on the conversion of oxaloacetate and acetyl-CoA to citrate, during which coenzyme A (CoASH) is released. CoASH subsequently reacts with 5,5′-dithiobis-(2-nitrobenzoic acid) (DTNB; Sigma-Aldrich) to form a coloured mercaptide ion [15]. Briefly, whole-cell extracts containing 20 µg of protein were added to a 150 μl reaction mixture containing 0.1 mM DTNB, 0.25% Triton X-100, 0.5 mM oxaloacetate, and 0.25 mg/ml acetyl-CoA in 0.1 M Tris buffer (pH 7.4). The optical density at 412 nm was measured after 3 minutes on an Immunoscan model 310 microplate reader (Labsystems, Finland).

### 2.4. Gas-liquid chromatography for fatty acid analysis

#### 2.4.1. Total lipid extraction

Total lipids were extracted using the Bligh and Dyer method [16]. Briefly, cell pellets were suspended in 1 mL distilled water and mixed with a chloroform:methanol (1:2, v/v) solution. After centrifugation at 3000 g for 5 minutes, the upper phase was transferred to a new tube. Chloroform and distilled water were added, followed by vigorous vortexing. After further centrifugation at 2000 rpm for 5 minutes, the lower chloroform phase containing lipids was collected for analysis.

#### 2.4.2. Separation of lipid fractions

The total lipid extract was dried under nitrogen gas and applied to silica gel thin-layer chromatography plates. Plates were developed in a solvent system consisting of hexane, diethyl ether, and glacial acetic acid (80:20:1, v/v/v) for 70 minutes. Lipid classes, phospholipids, triglycerides, and free fatty acids were identified by comparison with known standards based on their retention factor. Bands were scraped and transferred to screw-cap glass tubes for methylation.

#### 2.4.3. Fatty acid methyl ester derivatization

Fatty acid methyl esters (FAMEs) were prepared using the method modified from Lepage and Roy [17]. Briefly, lipid extracts were mixed with a methanol:hexane solution (4:1, v/v) containing 50 μg/mL tridecanoate as an internal standard. Acetyl chloride was added, and samples were sealed and incubated at 100°C for 1 hour. After derivatization, 6% potassium bicarbonate was added to neutralize the reaction, and the upper hexane layer containing esters was collected for analysis.

#### 2.4.4. Analysis of fatty acid methyl ester

FAMEs were analysed using gas – liquid chromatography on a Buck Scientific 610 system (SRI Instruments, USA) equipped with a TR-CN100 capillary column (60 m × 0.25 mm × 0.2 µm; Teknokroma, Spain) [12]. Helium was used as the carrier gas. The oven temperature was programmed to increase from 190°C to 210°C at a rate of 1°C/min, followed by a 20-minute isothermal hold at 210°C. A flame ionization detector was used to detect and quantify fatty acids, with retention times confirmed using a standard FAME mixture. Data was analysed using PeakSimple software.

### 2.5. Statistical analysis

All data are presented as mean ± standard deviation (SD). Fold changes were calculated as ratios relative to the reference group (MSCs or WLAs, as indicated). Reductions were reported as percentage changes, assuming the value in the reference group to be 100%. Statistical comparisons between groups were made using one-way analysis of variance (ANOVA), followed by Tukey’s post hoc test for multiple comparisons. Analyses were performed using GraphPad Prism software (GraphPad Software, USA). A p-value < 0.05 was considered statistically significant.

## 3. Results

### 3.1. Differentiation of human adipose MSCs into white-like and brown-like adipocytes

**Figure 1.**
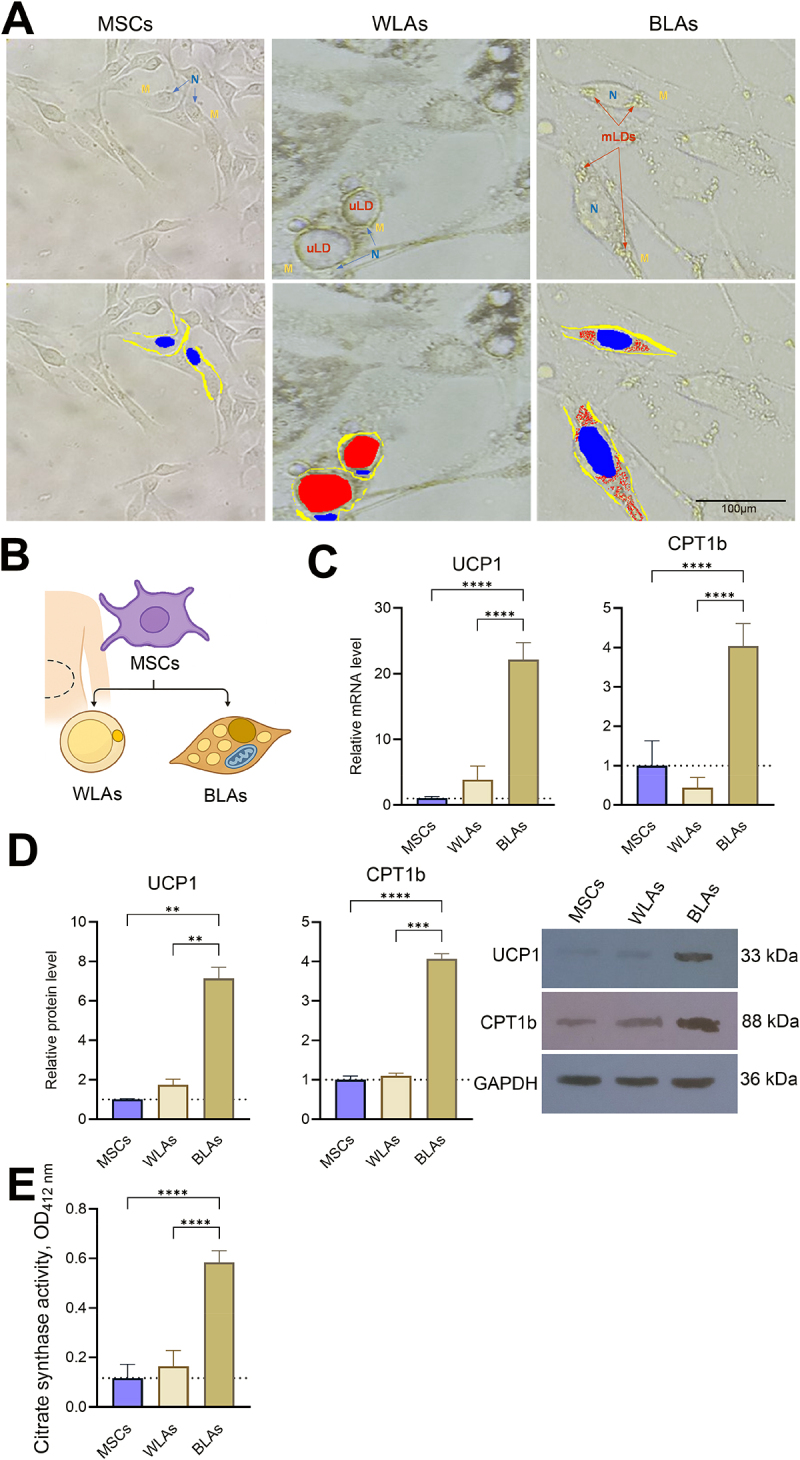
Differentiation of mesenchymal stem cells (MSCs) into white-like adipocytes (WLAs) and brown-like adipocytes (BLAs) in vitro. Phase-contrast microscopy images showing WLAs, and BLAs in culture (A). Schematic overview of experimental design (B). The top row shows phase-contrast microscopy images with labelled structures: nuclei (N), multilocular lipid droplets (mLDs), unilocular lipid droplets (uLDs), and the cell membrane (M). The bottom row presents pseudo-coloured versions, highlighting N (blue), mLDs (red), uLDs (red), and the M (yellow). mRNA (*n* = 4; C) and protein (*n* = 2; D) expression of uncoupling protein 1 (UCP1) and carnitine palmitoyltransferase 1b (CPT1b) after stimulation of MSCs with the adipogenic cocktail were measured by real-time polymerase chain reaction and Western blot analysis, respectively. Fold changes were calculated using MSCs as the control group. Citrate synthase activity was measured in cells extracts at 412 nm by detecting the reaction product formed during the enzyme activity (E). **p < 0.01, ****p < 0.0001.

To confirm the differentiation of human adipose-derived MSCs into WLAs and BLAs, we examined their morphology. Undifferentiated MSCs exhibited a typical fibroblast-like spindle shape (Figure 1A). Upon differentiation, WLAs developed large, unilocular lipid droplets, characteristic of white adipose cells. In contrast, BLAs displayed a morphology consistent with brown adipocytes, featuring multiple, smaller, multilocular lipid droplets.

To further validate brown adipocyte differentiation, we assessed UCP1 expression, as a key thermogenic marker, and CPT1b, as a central regulator of mitochondrial fatty acid oxidation, at both mRNA and protein levels. As shown in Figure 1C, quantitative real-time PCR revealed dramatic increases in UCP1 and CPT1b mRNA levels in BLAs compared with undifferentiated MSCs and WLAs (4–22-fold, *p* < 0.0001).

Consistent with the mRNA findings, Figure 1D demonstrates significantly higher UCP1 and CPT1b protein levels in BLAs, as determined by Western blot analysis. Relative quantification revealed ~ 7-fold higher UCP1 and ~ 4-fold higher CPT1b protein levels in BLAs compared with MSCs, and a significant ~ 4-fold increase for both proteins compared with WLAs (*p* < 0.01).

Citrate synthase, a key marker of mitochondrial activity, was also markedly elevated in BLAs, showing an approximate ~ 5-fold increase compared with both MSCs and WLAs (Figure 1E).

### 3.2. Distinct fatty acid composition in lipid fractions of white-like and brown-like adipocytes

**Figure 2.**
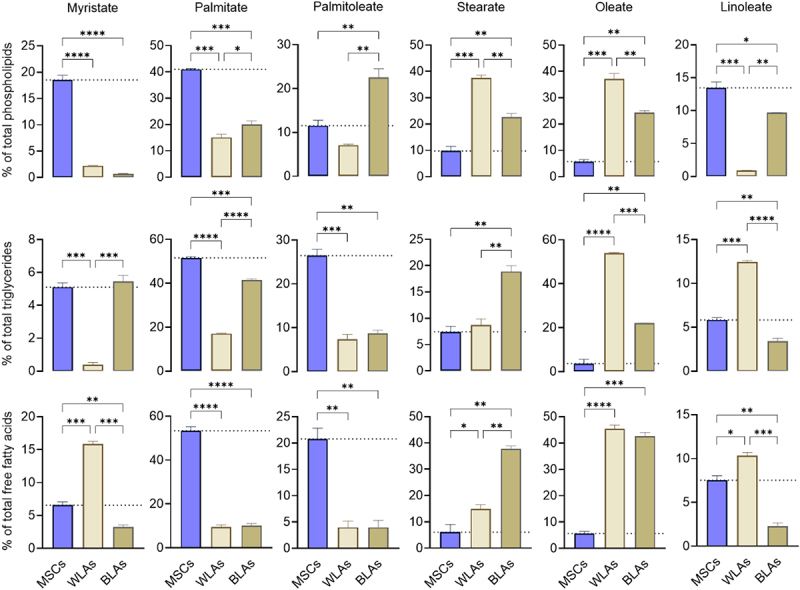
Fatty acid composition in phospholipid, triglyceride, and free fatty acid (FFA) fractions of white-like adipocytes (WLAs) and brown-like adipocytes (BLAs) differentiated from human adipose-derived mesenchymal stem cells (MSCs). The undifferentiated MSC group served as the control. Data are presented as mean ± SD (*n* = 2). *p < 0.05, **p < 0.01, ***p < 0.001, ****p < 0.0001.

We next investigated the fatty acid composition within the phospholipid, triglyceride, and free fatty acid fractions of undifferentiated MSCs, WLAs, and BLAs (Figure 2).

#### 3.2.1. Phospholipid fraction

In the phospholipid fraction, BLAs exhibited a significant reduction in myristate (−96%, *p* < 0.0001), palmitate (−51%, *p* < 0.001), and linoleate (−28%, *p* = 0.01) compared to undifferentiated MSCs. Conversely, levels of palmitoleate (2.0-fold), stearate (2.3-fold), and oleate (4.2-fold) were significantly elevated (*p* < 0.01 for all).

When comparing BLAs to WLAs, stearate (−39.6%) and oleate (−34.5%) were significantly lower (*p* < 0.01), while palmitate (1.3-fold, *p* = 0.03), palmitoleate (3.2-fold), and linoleate (10.8-fold) were significantly increased (*p* < 0.01).

Compared to MSCs, WLAs showed marked decreases in myristate (−88.0%, *p* < 0.0001), palmitate (−63.1%, *p* < 0.001), and linoleate (−93.3%, *p* = 0.01), along with robust increases in stearate (3.8-fold) and oleate (6.5-fold; *p* < 0.001 for both).

#### 3.2.2. Triglyceride fraction

In the triglyceride fraction, BLAs showed significantly reduced levels of palmitate (−19.6%, *p* < 0.001), palmitoleate (−66.9%, *p* < 0.01), and linoleate (−41.9%, *p* < 0.01) compared to MSCs. In contrast, stearate and oleate were markedly elevated by 2.5-fold and 6.1-fold, respectively (*p* < 0.01 for both).

When comparing BLAs to WLAs, oleate (−59%, *p* < 0.001) and linoleate (−72.8%, *p* < 0.0001) were significantly lower, whereas myristate (14.2-fold, *p* < 0.001), palmitate (2.4-fold, *p* < 0.001), and stearate (2.2-fold, *p* < 0.01) were significantly higher.

In WLAs compared to MSCs, triglyceride levels of myristate (−92.5%, *p* < 0.001), palmitate (−66.9%, *p* < 0.0001), and palmitoleate (−72.1%, *p* < 0.001) were significantly reduced.

#### 3.2.3. Free fatty acid fraction

In the free fatty acid fraction, BLAs showed significantly decreased levels of myristate (−51.1%, *p* < 0.01), palmitate (−81.1%, *p* < 0.0001), palmitoleate (−80.8%, *p* < 0.01), and linoleate (−69.5%, *p* < 0.01) compared to MSCs. In contrast, stearate (6.2-fold, *p* < 0.01) and oleate (7.6-fold, *p* < 0.001) were significantly increased.

Compared to WLAs, BLAs exhibited significantly lower levels of myristate (−79.7%) and linoleate (−77.7%) (*p* < 0.001), while stearate was elevated (2.5-fold, *p* < 0.01).

In WLAs versus MSCs, levels of palmitate (−82.2%, *p* < 0.0001) and palmitoleate (−80.8%, *p* < 0.01) were significantly decreased, whereas myristate (2.4-fold, *p* < 0.001), stearate (2.4-fold, *p* = 0.04), oleate (8.1-fold, *p* < 0.0001), and linoleate (1.4-fold, *p* = 0.01) were significantly increased.

### 3.3. Desaturation, elongation and ω6 synthesis indices of lipid fractions in WLAs and BLAs

**Figure 3.**
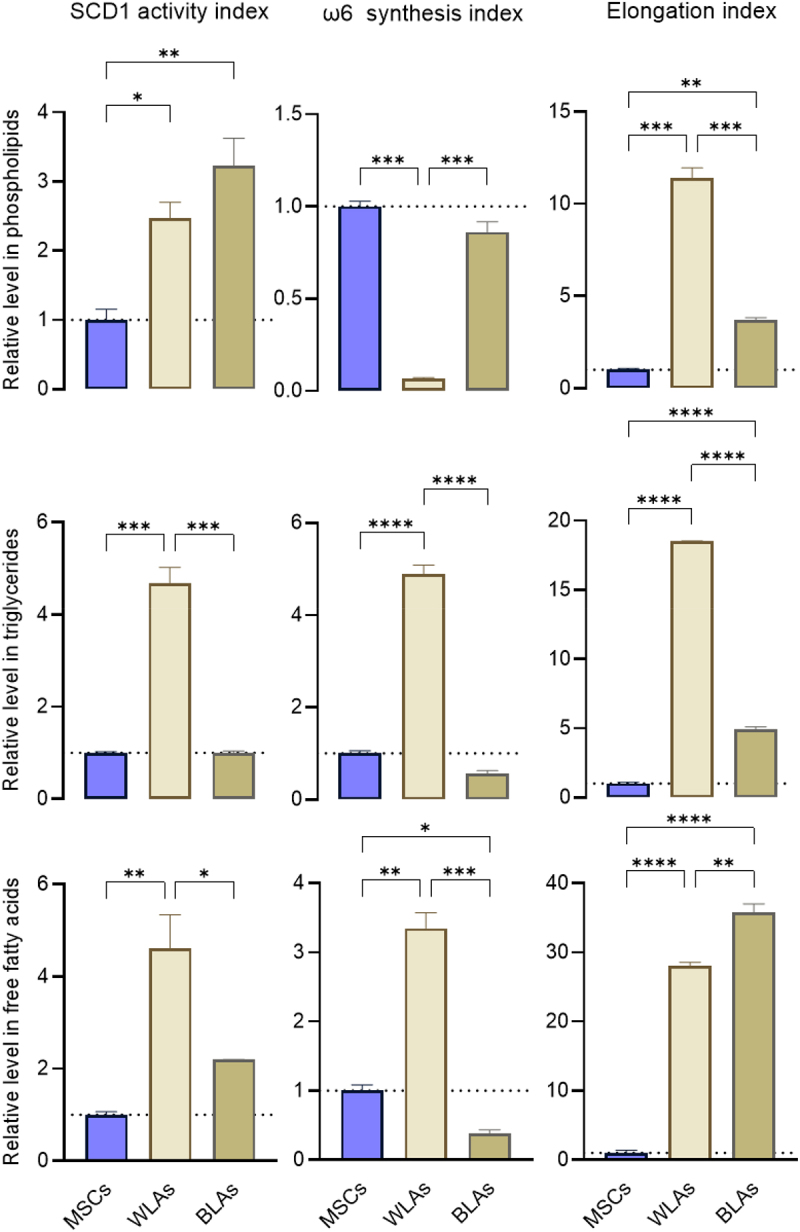
Stearoyl-CoA desaturase (SCD1) activity index, ω6 fatty acid synthesis index, and elongation index in phospholipid, triglyceride, and free fatty acid fractions in white-like adipocytes (WLAs) and brown-like adipocytes (BLAs) differentiated from human adipose-derived mesenchymal stem cells (MSCs). The undifferentiated MSC group served as the control. Data are presented as mean ± SD (*n* = 2). *p < 0.05, **p < 0.01, ***p < 0.001, ****p < 0.0001.

The percentages of individual fatty acids were used to calculate indices reflective of key enzymatic activities: stearoyl-CoA desaturase-1 (SCD1), elongation, and ω6 fatty acid synthesis (Figure 3). The SCD1 index was defined as (oleate + palmitoleate)/(stearate + palmitate), the elongation index as (stearate + oleate)/(palmitate + palmitoleate), and the ω6 synthesis index as linoleate/(stearate + palmitate).

#### 3.3.1. Phospholipid fraction

In the phospholipid fraction, BLAs showed significantly increased SCD1 activity (3.2-fold) and elongation index (3.7-fold, *p* = 0.01) compared to MSCs. When compared to WLAs, BLAs exhibited a marked reduction in elongation index (−67.2%) but a strong elevation in the ω6 synthesis index (13.2-fold, *p* < 0.001). WLAs versus MSCs showed elevated SCD1 activity (2.5-fold, *p* < 0.01) and elongation (11.4-fold, *p* < 0.001), whereas the ω6 synthesis index was significantly decreased (−93.5%, *p* < 0.001).

#### 3.3.2. Triglyceride fraction

In the triglyceride fraction, BLAs demonstrated a strong increase in elongation index (4.9-fold, *p* < 0.0001) relative to MSCs. Compared to WLAs, BLAs showed significant reductions in SCD1 activity (−78.5%, *p* < 0.001), elongation (−73.4%), and ω6 synthesis index (−88.7%) (*p* < 0.0001 for all). In contrast, WLAs compared to MSCs showed marked elevations in SCD1 activity (4.7-fold, *p* < 0.001), elongation (18.5-fold), and ω6 synthesis index (4.9-fold, *p* < 0.0001).

#### 3.3.3. Free fatty acid fraction

In the free fatty acid fraction, BLAs versus MSCs showed a significant decrease in ω6 synthesis index (−88.7%, *p* = 0.04), accompanied by a strong increase in elongation index (35.8-fold, *p* < 0.0001). Compared to WLAs, BLAs exhibited reduced SCD1 activity (−52.4%, *p* = 0.02) and ω6 synthesis (−88.7%, *p* < 0.001), while elongation index was modestly increased (1.3-fold, *p* < 0.01). In WLAs relative to MSCs, all three indices, SCD1 (4.6-fold, *p* < 0.01), elongation (28-fold, *p* < 0.0001), and ω6 synthesis (3.3-fold, *p* < 0.01), were significantly elevated.

## 4. Discussion

Although the fatty acid composition of phospholipid, free fatty acid, and triglyceride fractions is a major determinant of adipocyte function, little is known about the lipidomic characteristics of adipocytes derived from stem cells in vitro. In this study, we examined the fatty acid profiles of distinct lipid fractions after the differentiation of adipose-derived MSCs into WLAs and BLAs.

Differentiation into both adipocyte types was accompanied by significant alterations in the phospholipid fraction, notably an increase in stearate and oleate levels. These changes likely reflect membrane lipid remodelling that enhances structural stability and modulates membrane fluidity as properties critical for adipocyte signalling and function [18]. Furthermore, the phospholipid monolayer surrounding lipid droplets becomes enriched in monounsaturated fatty acids during differentiation, further supporting droplet expansion and dynamic lipid storage [7]. Concurrently, levels of myristate, palmitate, and linoleate decreased, likely facilitating membrane plasticity and creating a favourable environment for adipogenic maturation [19]. Both adipocyte types also exhibited significantly elevated desaturase and elongation indices relative to undifferentiated MSCs, supporting the notion that increased desaturation and elongation are integral to adipocyte maturation [19]. Previous studies have established that fatty acid desaturation influences lipid remodelling and inflammatory responses in adipose tissue [20,21]. Interestingly, only WLAs exhibited a marked reduction in the ω6 synthesis index compared to MSCs, suggesting a shift towards de novo lipogenesis and increased production of saturated fatty acids. This is in line with previous reports indicating that adipocyte differentiation is often associated with a reduction in polyunsaturated fatty acids and an increase in saturated and monounsaturated species [22,23].

In our human cell model, BLAs showed a thermogenically active phenotype characterized by enhanced lipid utilization and mitochondrial function relative to WLAs and MSCs. In the phospholipid fraction, relative to MSCs, BLAs displayed decreased levels of palmitate and linoleate, and increased palmitoleate, stearate and oleate, accompanied by a significant increase in desaturation index. When comparing BLAs to WLAs in the phospholipid fraction, BLAs exhibited elevated levels of palmitate, palmitoleate, and linoleate, with reduced stearate and oleate. These shifts suggest an adaptation in membrane fluidity that may be essential for supporting thermogenic function, as membrane composition can regulate the activity of heat-producing proteins [24]. Notably, BLAs also showed increased ω6 synthesis index but decreased elongation index compared to WLAs, reflecting a membrane remodelling strategy that favours fluidity and rapid signalling.

Compared to undifferentiated MSCs, the triglyceride fraction in BLAs was relatively enriched in 18-carbon fatty acids, but depleted in 16-carbon fatty acids and linoleate. This pattern suggests that BLAs either enhance fatty acid elongation or actively channel shorter-chain fatty acids into β-oxidation or membrane biosynthesis, rather than storing them in lipid droplets. These changes are consistent with previous reports linking fatty acid elongation to brown adipose tissue thermogenesis [25,26]

When directly comparing BLAs with WLAs, all lipid metabolic indices were significantly lower in BLAs, accompanied by elevated levels of saturated fatty acids. This pattern has been associated with reduced reliance on de novo synthesized unsaturated fatty acids and a diminished thermogenic capacity [27–29]. In contrast, WLAs showed increased lipogenesis indices compared to MSCs and BLAs, a lipid profile supportive of lipid droplet formation and energy storage typical of white adipocytes [30].

Upon the differentiation of MSCs into BLAs, the free fatty acid profile shifted towards higher levels of the long-chain saturated stearate and the monounsaturated fatty acid oleate, with lower levels of the polyunsaturated fatty acids linoleate. These changes may support mitochondrial function and thermogenesis [31]. In particular, dietary stearate enhanced mitochondrial fusion [32] and increased thermogenic capacity in brown adipose tissue [25]. High oleate may also help counteract the negative effects of the saturated fatty acid stearate, supporting cell viability and metabolic function [33]. This fatty acid remodelling suggests a metabolic shift favouring oxidation-tolerant and less lipotoxic fatty acids, consistent with the energy-expending phenotype of brown adipocytes. In contrast, WLAs showed elevated oleate levels, favouring lipid storage and survival under energy-rich conditions, hallmarks of white adipose tissue [34]. Furthermore, BLAs exhibited increased desaturation and elongation indices in the free fatty acid fraction, possibly reflecting a mitochondrial activation strategy. Prior studies have shown that long-chain fatty acids such as oleate and linoleate can increase basal metabolic rate and depolarize the inner mitochondrial membrane, potentially via a protonophoric mechanism [35,36].

In BLAs, SCD1 activity index was selectively elevated in phospholipids and free fatty acids, while remaining unchanged in triglycerides. This compartment-specific regulation suggests shifts in membrane composition and bioactive lipid pools, with potential consequences for fluidity and signalling, while leaving triglyceride-based energy storage largely unaffected. Thus, triglycerides remain enriched in saturated fatty acids which can be attributed to increased lipid turnover during thermogenic activation [37]. Such a dual effect aligns with observations in murine brown adipose tissue, where SCD1 appears dispensable for basal thermogenesis but critical under conditions of non-shivering thermogenesis, underscoring role in metabolic adaptability [38]. Overall, these findings underscore the functional divergence in lipid remodelling between brown and white adipocytes, aligning with their respective metabolic roles in energy expenditure versus storage.

Using cells from a single donor in this study is subject to donor-dependent heterogeneity and may not provide sufficient material for large-scale studies or applications [39]. Even within a single donor, MSCs can display variability in differentiation capacity, leading to inconsistent experimental outcomes [39]. Consequently, findings from single-donor studies may not fully translate to broader populations due to unique donor-specific traits. Nevertheless, this focused approach ensures genetic consistency and experimental control, allowing us to identify clinically relevant biological effects [40]. Thus, our data provides a foundation for future studies that incorporate interindividual variation.

## 5. Conclusion

This study highlights the distinct lipid profiles of WLAs and BLAs derived from human MSCs. While WLAs are enriched in long-chain monounsaturated fatty acids, favouring lipid storage, BLAs exhibit a more diverse fatty acid composition across lipid fractions associated with greater membrane fluidity, metabolic flexibility, and thermogenic potential. These lipidomic distinctions advance our understanding of adipocyte heterogeneity and may inform future therapeutic strategies targeting adipose tissue in obesity and metabolic diseases. In particular, approaches that modulate lipid remodelling – such as targeting desaturases activity or enhancing mitochondrial plasticity – could help improve metabolic health.

## Supplementary Material

Supplemental Material

## Data Availability

Data supporting the findings of this study, including fatty acid composition data, uncropped Western blots, and experimental protocols for MSC isolation and lipid analysis, were deposited on Figshare at https://doi.org/10.6084/m9.figshare.29561339).
