# Supplementary material for: Fatty acid composition of lipid fractions in white- and brown-like adipocytes derived from human mesenchymal stem cells
Source: Adipocyte. 2025 Oct 1;14(1):2566481. doi: 10.1080/21623945.2025.2566481 (PMC12498536; doi:10.1080/21623945.2025.2566481)
Supplement: Supplemental Material [file KADI_A_2566481_SM3465.docx]

**Supplementary Table 1.** PCR primer sequences for real-time PCR analysis.

| **Gene** | **Accession number** | **Direction** | **Sequence (5' to 3')** | **Product size (bp)** |
| --- | --- | --- | --- | --- |
| UCP1 | NM_021833 | forward | GGAGGAGTGGCAGTATTCAT | 165 |
|  |  | reverse | AGTAGTCCCTTTCCAAAGACC |  |
| CPT1b | NM_152246 | forward | GTATCGCCGTAAACTGGACCG | 146 |
|  |  | reverse | TGTCTGAGAGGTGCTGTAGCAC |  |
| GAPDH | NM_002046 | forward | TTGGCTACAGCAACAGGGTG | 161 |
|  |  | reverse | GGGGAGATTCAGTGTGGTGG |  |
